# The Periphery of Salivary Gland Carcinoma Tumors Reveals a PD-L1/PD-1 Biomarker Niche for the Evaluation of Disease Severity and Tumor—Immune System Interplay

**DOI:** 10.3390/biomedicines9020097

**Published:** 2021-01-20

**Authors:** Martin Kuchar, Zuzana Strizova, Linda Capkova, Martin Komarc, Jiri Skrivan, Jirina Bartunkova, Daniel Smrz, Jan Plzak

**Affiliations:** 1 Department of Otorhinolaryngology and Head and Neck Surgery, First Faculty of Medicine, Charles University and University Hospital Motol, 15006 Prague, Czech Republic; martin.kuchar@fnmotol.cz (M.K.); jan.plzak@fnmotol.cz (J.P.); 2 Department of Immunology, Second Faculty of Medicine, Charles University and University Hospital Motol, 15006 Prague, Czech Republic; jirina.bartunkova@fnmotol.cz (J.B.); daniel.smrz@fnmotol.cz (D.S.); 3 Department of Pathology and Molecular Medicine, Second Faculty of Medicine, Charles University and University Hospital Motol, 15006 Prague, Czech Republic; linda.capkova@fnmotol.cz; 4 Department of Methodology, Faculty of Physical Education and Sport, Charles University, 16252 Prague, Czech Republic; komarc@volny.cz; 5 Department of Otorhinolaryngology, Second Faculty of Medicine, Charles University and University Hospital Motol, 15006 Prague, Czech Republic; jiri.skrivan@fnmotol.cz

**Keywords:** salivary gland carcinoma, PD-L1, PD-1, tumor periphery, tumor center, grade, stage, lymph node metastases

## Abstract

The treatment options for patients with advanced salivary gland cancers (SGCs) are limited. Immune checkpoint inhibitors (ICIs) have revolutionized cancer treatment. However, the response to ICI immunotherapy is largely driven by the immune cell signatures within the tumor tissue and the para-tumoral tissue compartments. To date, there are no data on the expression of programed cell death protein-1/programed cell death protein-ligand 1 (PD-1/PD-L1) in SGC, which may enable the implementation of ICI immunotherapy for this disease. Thus, we performed an immunohistochemical analysis of PD-1 and PD-L1 expression in tumor cells and tumor-infiltrating immune cells (TIICs) in the tumor center and periphery of 62 SGC patients. The tumor periphery showed significantly higher expression of PD-L1 in tumor cells than in TIICs. Moreover, peripheral TIICs had significantly higher PD-1 expression than peripheral tumor cells. PD-1-positive tumor cells were detected exclusively in the tumor center of high-grade tumors, and most importantly, the presence of lymph node (LN) metastases and primary tumor stage significantly correlated with the presence of PD-L1-positive tumor cells in the tumor periphery. The PD-1/PD-L1 molecular signatures in SGC are clustered predominantly in the tumor periphery, reflect disease severity, and may predict the response to ICI immunotherapy in SGC patients.

## 1. Introduction

Salivary gland cancers (SGCs) represent a rare group of neoplasms, accounting for less than 5% of head and neck cancers [1]. According to the World Health Organization (WHO) classification, more than 30 histological subtypes of salivary gland tumors have been identified, and 24 of them are proposed as malignant [2]. Due to the rarity of these cancers and their extreme histological diversity, defining the optimal molecular targets for SGC treatment is extremely challenging [3]. Whereas early stages of SGC can be effectively treated by surgery, patients with unresectable, recurrent, and metastatic (advanced) SGCs have limited treatment options and, thus, a poor prognosis [3,4]. In addition, conventional oncologic regimens, such as radiotherapy and chemotherapy, have low efficacy and are associated with severe adverse events, including neutropenia, xerostomia, dysphagia, and neurological complications [5,6,7].

A number of novel immunotherapeutic and targeted therapeutic strategies have been implemented in clinical practice to treat advanced and metastatic cancers [8,9]. In SCGs, previous studies have revealed new routes to targeted therapies by uncovering the most common and clinically relevant genetic aberration, such as the *TP53* gene, cyclin pathway, or PI3K pathway alterations [10,11]. Among the immunotherapeutic strategies, checkpoint inhibitors represent the major breakthrough in the treatment of these cancers [9]. The most promising results have been achieved with immune checkpoint inhibitors (ICIs) targeting cytotoxic T-lymphocyte-associated protein 4 (CTLA-4) (ipilimumab, tremelimumab), programmed cell death (PD)-1 (nivolumab, pembrolizumab, and cemiplimab), and PD-ligand 1 (PD-L1) (atezolizumab, avelumab, and durvalumab) [12]. However, even though ICIs have revolutionized cancer treatment, a significant percentage of patients remain resistant to immunotherapy. This resistance is elicited through multiple mechanisms, including low infiltration with tumor-infiltrating immune cells (TIICs) or limited expression of checkpoint molecules and their ligands in tumor cells and/or TIICs [13,14]. Multiple studies have attempted to find algorithms to predict the efficacy of ICI immunotherapy based on the expression of these molecules in tumor cells and TIICs [14]. However, only a few studies have investigated the expression of checkpoint molecules in the selected histological subtypes of SGC [15,16,17,18,19].

In this study, we examined the expression of PD-L1 and PD-1 in tumor cells and TIICs in the center and periphery of primary tumors from 62 SGC patients with 13 different histological subtypes. The data obtained were correlated with the patients’ clinicopathological data to evaluate how disease severity shapes the tumor expression landscape of checkpoint molecules to determine new biomarkers for the implementation of ICI immunotherapy in SGCs.

## 2. Materials and Methods

### 2.1. Patients

In this retrospective, single-center study, we investigated the expression of PD-1 and PD-L1 on the cell surface of tumor cells and tumor-infiltrating lymphocytes in SGC patients. All patients with SGC (*n* = 62) underwent surgery between January 2013 and December 2018 and provided written consent to participate in the study. A total number of 36 females and 26 males with the age range from 21 to 85 years were included (Table 1). All patients were Caucasian. All experimental protocols were approved by the Ethical Standards of the Institutional and/or National Research Committee—the Ethics Committee of the University Hospital Motol in Prague (EK-1394/20; approved 23 October 2020)—and performed in accordance with the 1964 Helsinki declaration and its later amendments or comparable ethical standards. A total of 13 histological subtypes were included. The study cohort consisted of 8 acinic cell carcinomas, 11 adenoid cystic carcinomas, 6 adenocarcinomas, not otherwise specified (NOS), 1 adenosquamous carcinoma, 1 carcinosarcoma, 3 carcinoma expleiomorphic adenomas, 4 undifferentiated carcinomas, 6 salivary duct carcinomas, 1 cribriform cystadenocarcinoma, 3 mammary analogue secretory carcinomas (MASCs), 13 mucoepidermoid carcinomas, 3 myoepithelial carcinomas, and 2 squamous cell carcinomas. Each tumor was scored based on tumor grade and stage. Tumor grade was determined according to the histopathological evaluation and with respect to the risk stratification of salivary gland malignancies established by the WHO [20]. Tumors were classified as either low grade (grade 0) or high grade (grade 1). Primary tumor staging was carried out using the 8th edition of the Union of International Cancer Control/American Joint Committee on Cancer UICC/AJCC TNM (tumor, node, metastasis) staging system for head and neck cancer [21]. In one patient, the stage of the primary tumor could not be established. In each patient, the presence of regional LN metastases was evaluated by ultrasound or magnetic resonance imaging and correlated with histopathological data.

### 2.2. Sample Preparation and Immunohistochemistry

Patient tissue samples were formalin-fixed and paraffin-embedded (FFPE) in the University Hospital Motol. The histologic sections were retrospectively retrieved and analyzed with approval from the local ethics committee (EK no. EK-1394/20). The diagnosis and reliability of the slides were reviewed by a well-experienced pathologist, and each tissue sample was scored manually. To quantify the staining results and avoid subjective bias, the intra-rater reliability test was performed. Slides (3 µm thick) were stained using the following prediluted antibodies: anti-PD-L1 (Cat no. M3653; Dako, Santa Clara, CA, USA) and anti-PD-1 (CD279) (Cat no. 516-18662; Zytomed, Berlin, Germany). PD-1 and PD-L1 expression on tumor and immune cells was analyzed separately. Furthermore, the expression of PD-1/PD-L1 was measured in the tumor center as well as in the tumor periphery. The tumor periphery was defined as the area covering the tumor–host interface extending one high-power field (HPF, 400-fold magnification) from the outline of the tumor. The tumor center was defined as the area extending from the tumor–host interface towards the tumor center [22]. The proportion of positive cells was assessed according to the tumor proportion score (TPS), which is widely used in the head and neck tumors and was shown to serve as a valuable marker for the prediction of ICI response [23,24,25]. Within this approach, only strong PD-1 cytoplasmic staining of TIIC/tumor cells was regarded as a significant staining, and strong PD-L1 membranous staining of tumor cells and strong PD-L1 membranous and cytoplasmic staining of TIIC were regarded as positive staining (Table 2). The following method was applied: TPS = (No. of PD-1 or PD-L1 stained tumor cells)/(total No. of viable tumor cells) × 100; (No. of PD-1 or PD-L1 stained tumor-infiltrating immune cells)/(total No. of viable tumor-infiltrating immune cells) × 100; No. = cell count.

In accordance with previously published PD-1/PD-L1 studies, the proportions of positive cells (assessed by TPS) were scored as follows: score 0: negative, score 1 (weak): 1–10%, score 2 (moderate): 10–49%, and score 3 (strong): above 50% [26,27,28,29]. The detailed scoring system was translated into heatmaps (Figures 3–5) where the color scale was presented as follows: yellow color: score 0, marigold color: score 1, ochre color: score 2, and bronze color: score 3. In cases where lymphocyte infiltration was not found, PD-1/PD-L1 expression was classified as N/A (not applicable).

### 2.3. Statistical Analysis

The data are presented as the mean ± standard error of mean (SEM) unless otherwise stated. Bivariate associations between studied variables were assessed by Spearman’s rank-order correlation coefficient. The Mann–Whitney U test was performed to assess differences in PD-1 and PD-L1 expression based on different grouping variables (grade: 0/1; regional LN metastases: No/Yes; primary tumor stage: 1, 2, 3, and 4). Differences in paired measurements (e.g., PD-1 in the tumor center/PD-1 in the tumor periphery; PD-L1 in TIICs/PD-L1 in tumor cells) were tested by the Wilcoxon signed-rank test. Both Mann–Whitey U and Wilcoxon tests were conducted using the Monte Carlo resampling procedure with *n* = 10,000 samples, which compensate for tied values and do not depend on asymptotic approximations for *p*-values. Statistical significance was tested at the α = 0.05 level (in some cases, we report *p*-values as follows: * p < 0.05, ** p < 0.01, *** p < 0.001, and **** p < 0.0001). SPSS Statistical Software version 25.0 (SPSS, Chicago, IL, USA) and GraphPad Prism 6 (GraphPad Software, La Jolla, CA) were used for statistical analyses.

## 3. Results

### 3.1. The Presence of Regional Lymph Node (LN) Metastases Significantly Correlates with the Tumor Grade and Primary Tumor Stage

In this study, we evaluated tumor samples from 62 SGC patients to provide deeper insight into this rare group of malignancies. As shown in Table 1, the 62 SGC tumor samples comprised 13 distinct histological subtypes. The most frequent diagnoses were acinic cell carcinoma, adenoid cystic carcinoma, and mucoepidermoid carcinoma. The least frequent were cribriform cystadenocarcinoma, adenosquamous carcinoma, and carcinosarcoma. Of the 62 SGC patients, 15 were diagnosed with primary tumor stage 1, 9 with stage 2, 6 with stage 3, and 31 with stage 4. In one patient, the primary stage of the tumor was not determined. Twenty-two patients were diagnosed with low-grade (grade 0) tumors, and forty patients were diagnosed with high-grade (grade 1) tumors. The presence of lymph node (LN) metastases was assessed in each patient.

**Figure 1:**
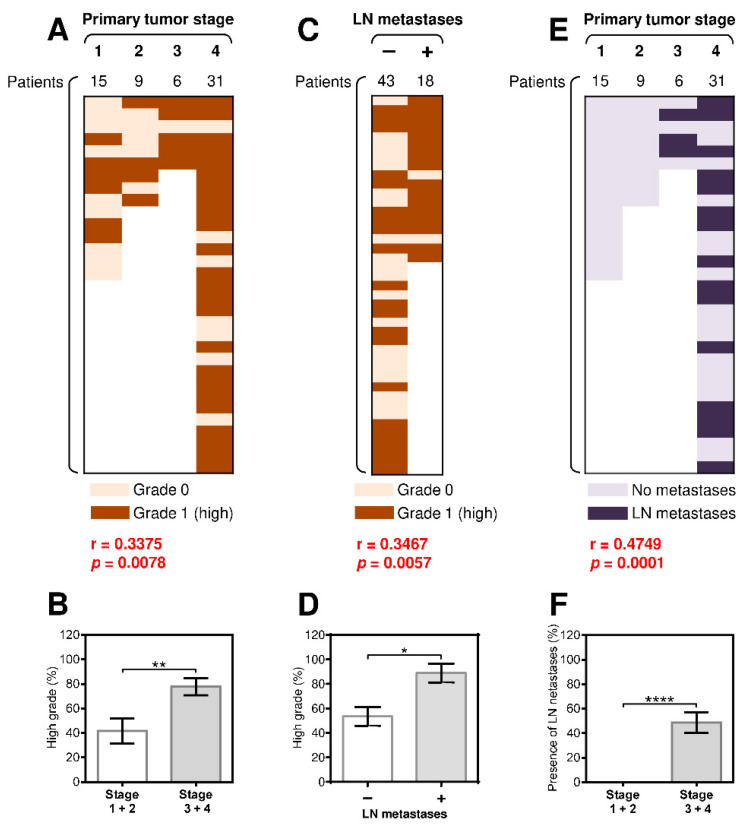
The presence of regional lymph node (LN) metastases significantly correlates with tumor grade and the primary tumor stage. (**A**) The cohort of 61 salivary gland cancer (SGC) patients was stratified into 4 groups according to the primary tumor stage, and the Spearman correlation with tumor grade (grade 0: light brown, grade 1: dark brown) was determined. (**B**) The patient cohort in (**A**) was stratified into 2 groups according to the primary tumor stage (group 1: stage 1 and 2 patients, group 2: stage 3 and 4 patients), and the proportion of high-grade tumors in each group was determined. (**C**) The patient cohort in (**A**) was stratified into 2 groups according to the presence (**+**) or absence (**–**) of LN metastases, and the correlation with the primary tumor stage was determined as in (**A**). (**D**) The proportion of high-grade tumors in each group in (**C**) was determined. (**E**) The patient cohort in (**A**) was stratified into 4 groups according to the primary tumor stage, and the correlation with LN metastases (no LN metastases: light blue, grade 1: dark blue) was determined. (**F**) The patient cohort in (**E**) was stratified into 2 groups according to the primary tumor stage (group 1: stage 1 and 2 patients, group 2: stage 3 and 4 patients), and the proportion of patients with LN metastases in each group was determined. In (**A**), (**C**), and (**E**), the correlations were evaluated by Spearman correlation tests (*n* = 61). * *p* < 0.05 was considered significant. In (**B**), (**D**), and (**F**), the difference between the stratified groups of patients was evaluated by the Mann–Whitney U test (stages 1 and 2, *n* = 24; stages 3 and 4, *n* = 37; * *p* < 0.05, ** p < 0.01, *** p < 0.001, **** p < 0.0001). The data are shown as the mean ± standard error of mean (SEM).

We first analyzed the association between the PD-1/PD-L1 expression among different age and gender groups, however, we did not observe any significant differences. Furthermore, we examined correlations between tumor grade and the primary tumor stage in SGC patients. Similar to studies of other cancer types [30,31], we found a significant correlation between tumor grade and the primary tumor stage (Figure 1A) and a significantly higher prevalence of high-grade tumors in primary stage 3 and 4 SGCs than in primary stage 1 and 2 SGCs (Figure 1B). We next analyzed whether disease severity also correlated with the presence of LN metastases. As shown in Figure 1C, there was a strong correlation between the presence of LN metastases and tumor grade. High-grade tumors were also more prevalent in patients with LN metastases than in patients without LN metastases (Figure 1D). An even stronger correlation was found between the presence of LN metastases and the primary tumor stage (Figure 1E–F). These data showed that the pathologically assessed severity of the disease (primary tumor stage and grade) in SGC patients also correlated with the metastatic potential of the disease.

### 3.2. The Differences between the Expression of PD-L1 and PD-1 in Tumor Cells and TIICs Are Clustered in the Tumor Periphery

**Figure 2:**
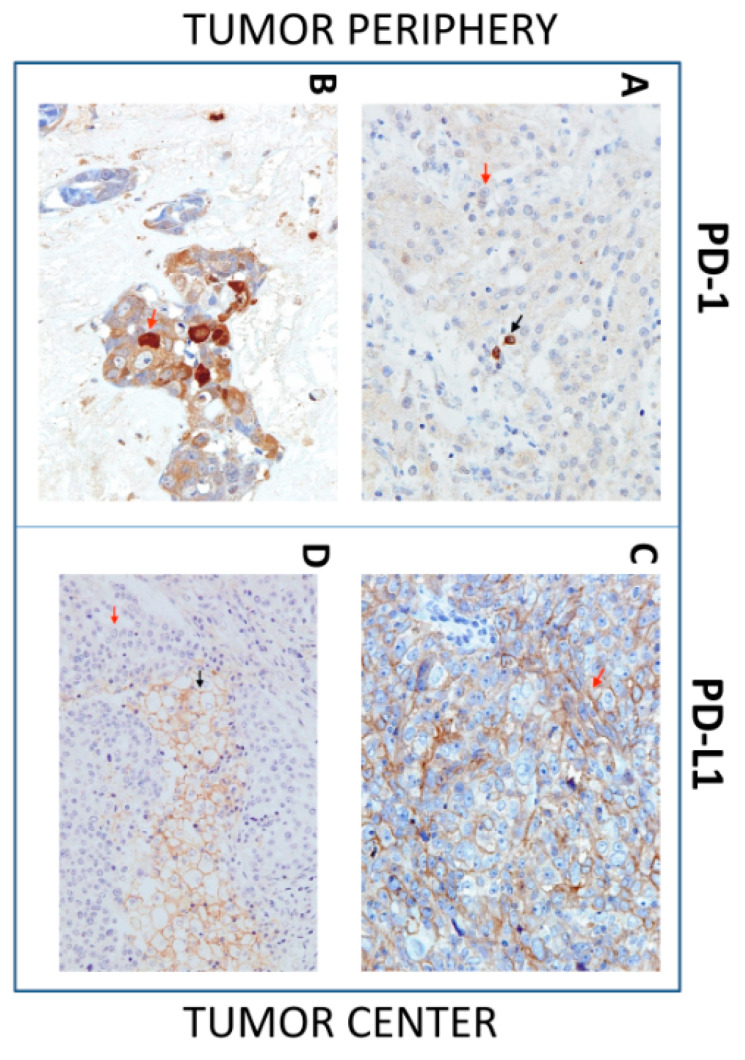
Immunohistochemistry (IHC) of Programmed cell death protein-1(PD-1) and Programmed cell death protein-1 ligand (PD-L1) expression in SGC tissues. Representative images show PD-1 or PD-L1 expression in tumor cells (red arrow) and tumor-infiltrating immune cells (TIICs), (black arrow) in the tumor periphery (left images) and tumor center (right images). (**A**) IHC shows positive PD-1 expression in TIICs and negative PD-1 expression in tumor cells (40×). (**B**) IHC shows positive PD-1 expression in tumor cells (40×). (**C**) IHC shows positive PD-L1 expression in tumor cells (40×). (**D**) IHC shows PD-L1-positive TIICs and PD-L1-negative tumor cells (40×).

Many studies have attempted to characterize the tumor microenvironment in multiple cancer types to reveal the potential for immunotherapy based on the expression of PD-L1 and PD-1 in tumors [14,32,33,34]. Studies have also shown that tumor-induced immunosuppression can be unevenly distributed within the tumor microenvironment [35,36]. Therefore, we examined the expression patterns of PD-L1 and PD-1 in tumor cells and TIICs in both the tumor center and periphery (Figure 2) using the scoring system detailed in the Materials and Methods Section.

**Figure 3:**
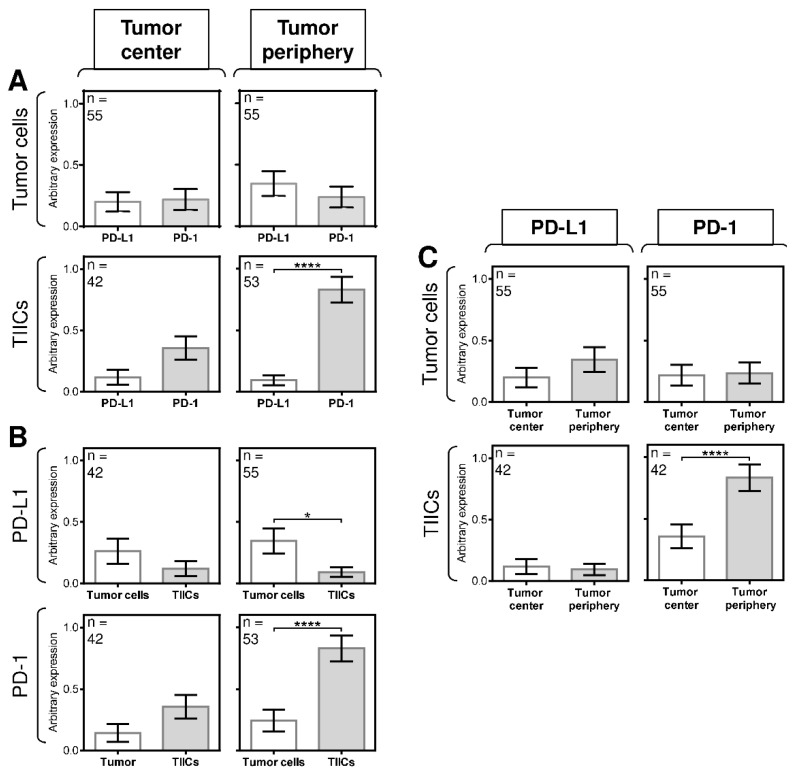
The differences between the expression of PD-L1 and PD-1 in tumor cells and TIICs are clustered in the tumor periphery. (**A**) The expression of PD-L1 and PD-1 differs between tumor cells and TIICs in the tumor center and tumor periphery. The number of patients (*n*) evaluated is shown in the top left corner of each panel. (**B**) The expression of PD-L1 and PD-1 differs between tumor cells and TIICs in the tumor center and tumor periphery. The number of patients (*n*) evaluated is shown in the top left corner of each panel. (**C**) The expression of PD-L1 and PD-1 differs between tumor cells and TIICs in the tumor center and tumor periphery. The number of patients (*n*) evaluated is shown in the top left corner of each panel. In (**A**–**C**), the difference between groups was evaluated with respect to the frequencies of the PD-1/PD-L1 expressions and their mean values and analyzed by the Mann–Whitney U test (*n* is shown in the top left corner of each panel; * p < 0.05, ** p < 0.01, *** p < 0.001, **** p < 0.0001). The data are shown as the mean ± SEM, and the arbitrary expression level corresponds to the scoring system described in the Materials and Methods Section.

We initially found no significant difference in the expression of PD-1 or PD-L1 in tumor cells and TIICs in the tumor center (Figure 3A, left panels) or in the peripheral tumor cells (Figure 3A, right top panel). However, the expression of PD-1 was significantly higher than the expression of PD-L1 in the peripheral TIICs (Figure 3A, right bottom panel). Further analyses revealed that differences in the expression of both molecules between tumor cells and TIICs also clustered in the tumor periphery. Whereas the tumor center contained tumor cells and TIICs with comparable expression of PD-L1 (Figure 3B, left top panel), the tumor periphery showed significantly higher expression of the molecule in the tumor cells than in TIICs (Figure 3B, right top panel). Similarly, no significant difference in the expression of PD-1 was observed between tumor cells and TIICs in the tumor center (Figure 3B, left bottom panel). However, the peripheral TIICs had significantly higher expression of PD-1 than the peripheral tumor cells (Figure 3B, right bottom panel).

These data indicated that the expression of PD-L1 and PD-1 in the cells in the tumor periphery was distinct from that in the tumor center. However, despite these indications, the next analyses showed no significant differences in the expression of PD-L1 between the tumor periphery and tumor center (Figure 3C, left panels), nor was there a difference in the expression of PD-1 between the tumor periphery and tumor center (Figure 3C, right top panel). However, a significant difference in the expression of PD-1 was found between the TIICs in the tumor periphery and tumor center (Figure 3C, right bottom panel). These data showed that the differences in the expression of PD-L1 and PD-1 in SGC patients were clustered in the tumor periphery.

### 3.3. Disease Severity Does Not Correlate with the Expression of PD-L1 and PD-1 in TIICs

**Figure 4:**
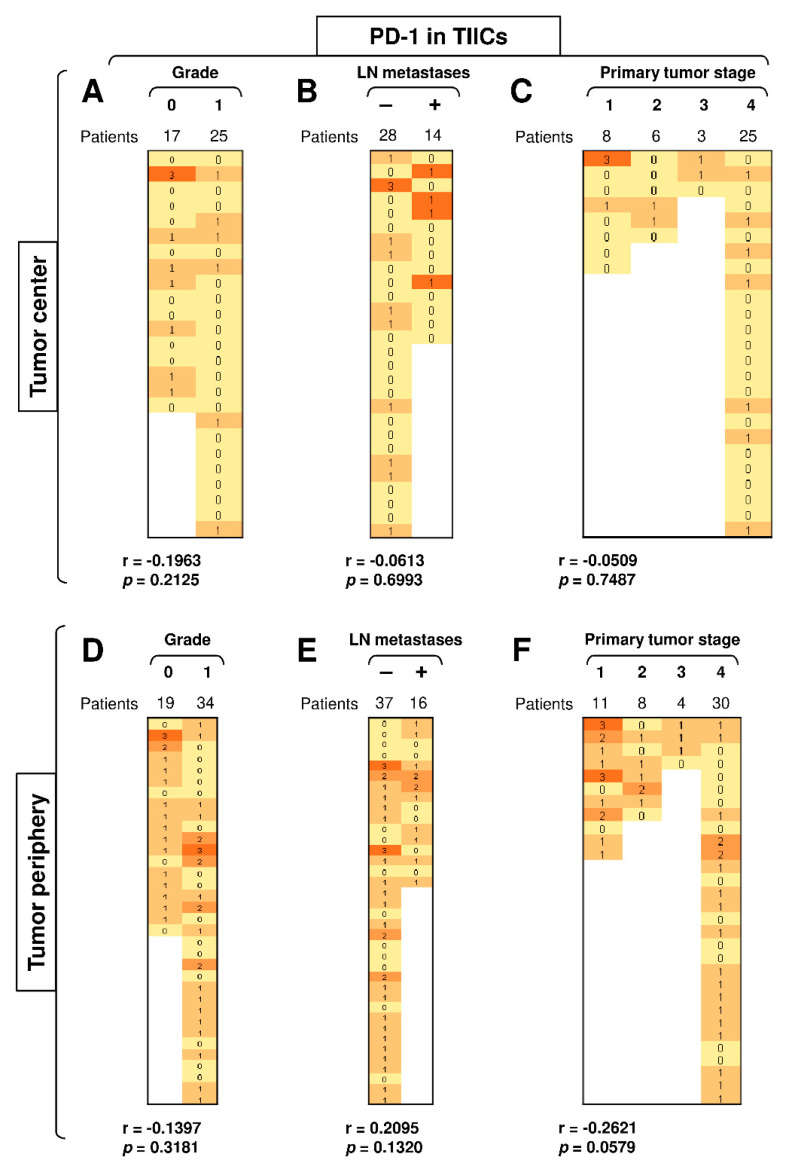
Disease severity does not correlate with the expression of PD-L1 and PD-1 in TIICs. (**A**) The cohort of 42 SGC patients was stratified into 2 groups according to tumor grade (grade 0 and grade 1), and the Spearman correlation with the expression of PD-1 in TIICs in the tumor center was determined. (**B**) The patient cohort in (**A**) was stratified into 2 groups according to the presence (**+**) or absence (**–**) of LN metastases, and the Spearman correlation was determined as in (**A**). (**C**) The patient cohort in (**A**) was stratified into 4 groups according to the primary tumor stage, and the Spearman correlation was determined as in (**A**). (**D**–**F**) The cohort of 53 SGC patients was stratified into groups as in (**A**–**C**), and the Spearman correlation with the expression of PD-1 in TIICs in the tumor periphery was determined. In (**A**–**F**), the correlations were evaluated by Spearman correlation tests, (**A**–**C**): *n* = 42, (**D**–**F**): *n* = 53. * p < 0.05 was considered significant. In (**A**–**F**), the expression analyses of PD-1 were performed according to the scoring system described in the Materials and Methods Section. The data are presented as a heat map with the scores. The color scale is presented as follows: yellow color: score 0; marigold color: score 1; ochre color: score 2; bronze color: score 3.

The expression of PD-1 and/or PD-L1 in multiple tumors often correlates with disease severity [37]. These molecules are also important biomarkers for the indication of ICI immunotherapy [9,13,14]. Since our data showed that the differences in the expression of PD-L1 and PD-1 in tumor cells and TIICs were clustered in the periphery of SGC patients’ tumors, we next evaluated whether this clustering also correlated with disease severity (tumor grade, primary tumor stage, and the presence of LN metastases). We found that disease severity did not correlate with the expression of PD-L1 in TIICs in either the tumor periphery or tumor center (Supplementary Figure S1), nor were there correlations between the abundantly expressed PD-1 in the TIICs in the tumor center (Figure 4A–C) or even in the much more abundantly expressed PD-1 in the peripheral TIICs (Figure 4D–F). These data showed that, although PD-1 expression was higher in the peripheral TIICs than in the central TIICs, this expression did not reflect disease severity.

### 3.4. A High Load of PD-1^+^ Tumor Cells in the Tumor Center Correlates with Tumor Grade

**Figure 5:**
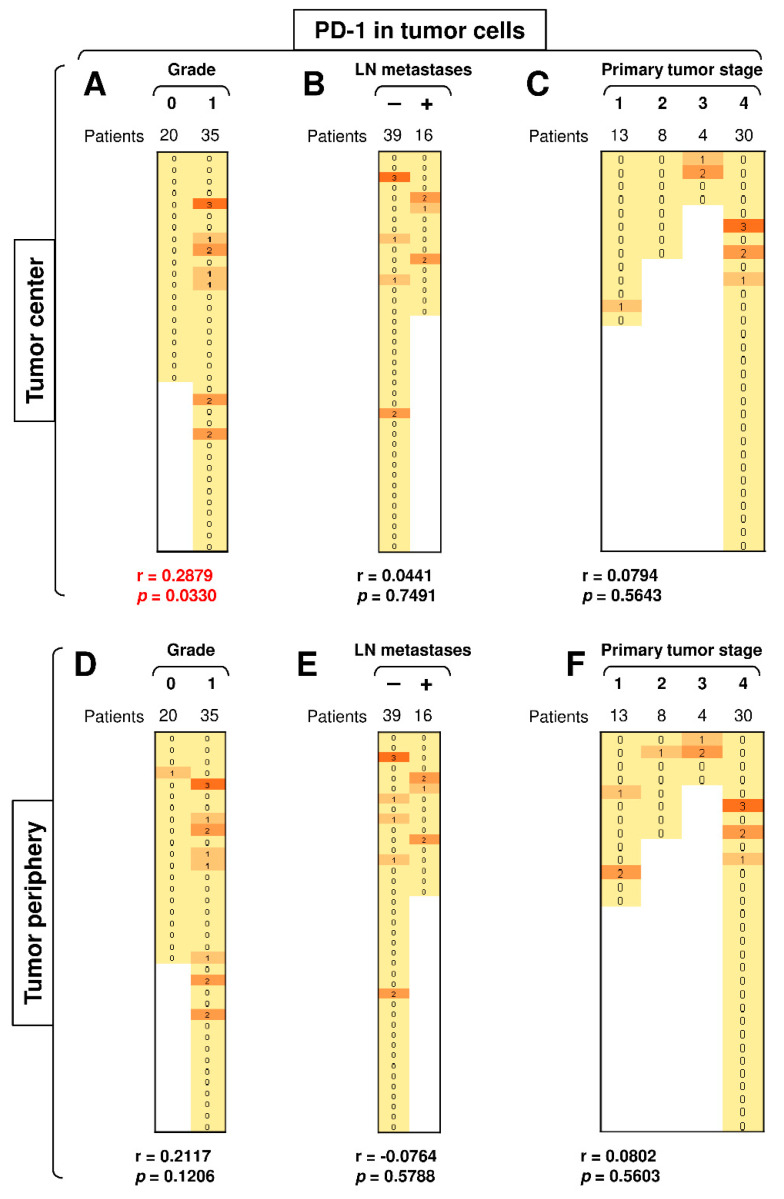
A high load of PD-1^+^ tumor cells in the tumor center correlates with tumor grade. (**A**) The cohort of 55 SGC patients was stratified into 2 groups according to tumor grade (grade 0 and grade 1), and the Spearman correlation with the expression of PD-1 in tumor cells in the tumor center was determined. (**B**) The patient cohort in (**A**) was stratified into 2 groups according to the presence (**+**) or absence (**–**) of LN metastases, and the Spearman correlation was determined as in (**A**). (**C**) The patient cohort in (**A**) was stratified into 4 groups according to the primary tumor stage, and the Spearman correlation was determined as in (**A**). (**D**–**F**) The cohort of 55 SGC patients was stratified into groups as in (**A**–**C**), and the Spearman correlation with the expression of PD-1 in tumor cells in the tumor periphery was determined. In (**A**–**F**), the correlations were evaluated by Spearman correlation tests, (**A**–**F**): *n* = 55. * *p* < 0.05 was considered significant. In (**A**–**F**), the expression analyses of PD-1 were performed according to the scoring system described in the Materials and Methods Section. The data are presented as a heat map with the scores. The color scale is presented as follows: yellow color: score 0; marigold color: score 1; ochre color: score 2; bronze color: score 3.

PD-1 is mostly expressed in T cells [38]. However, PD-1 can also be expressed in tumor cells [39]. Our analysis revealed that several tumors from SGC patients also expressed this molecule in both the central and peripheral tumor cells (Figure 5). Disease severity (tumor grade, primary tumor stage, and the presence of LN metastases) had no significant impact on the expression of this molecule in peripheral tumor cells (Figure 5D–F), nor did the presence of LN metastases or the primary tumor stage impact its expression in central tumor cells (Figure 5B–C). However, tumor grade was significantly associated with the PD-1 expression in the cells located in the tumor center because the PD-1 expression in the central tumor cells was detected exclusively in high-grade (grade 1) tumors but not in low-grade (grade 0) tumors (Figure 5A). These findings showed that the proportion of PD-1^+^ tumor cells in the tumor center could serve as a biomarker of disease severity.

### 3.5. The Presence of LN Metastases and Tumor Stage Significantly Correlate with PD-L1 Expression in Peripheral Tumor Cells

**Figure 6:**
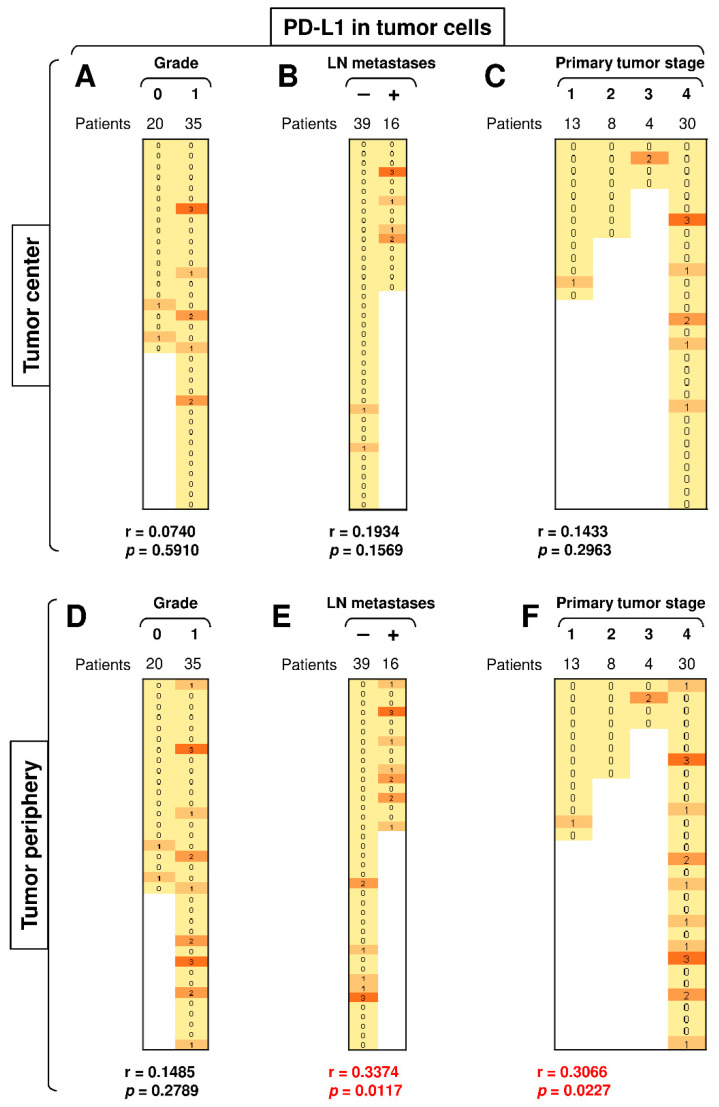
The presence of LN metastases and primary tumor stage significantly correlate with PD-L1 expression in peripheral tumor cells. (**A**) The cohort of 55 SGC patients was stratified into 2 groups according to tumor grade (grade 0 and grade 1), and the Spearman correlation with the expression of PD-L1 in tumor cells in the tumor center was determined. (**B**) The patient cohort in (**A**) was stratified into 2 groups according to the presence (+) or absence (–) of LN metastases, and the Spearman correlation was determined as in (**A**). (**C**) The patient cohort in (**A**) was stratified into 4 groups according to the primary tumor stage, and the Spearman correlation was determined as in (**A**). (**D**–**F**) The cohort of 55 SGC patients was stratified into groups as in (**A**–**C**), and the Spearman correlation with the expression of PD-L1 in tumor cells in the tumor periphery was determined. In (**A**–**F**), the correlations were evaluated by Spearman correlation tests, (**A**–**F**): *n* = 55. * p < 0.05 was considered significant. In (**A**–**F**), the expression analyses of PD-1 were performed according to the scoring system described in the Materials and Methods Section. The data are presented as a heat map with the scores. The color scale is presented as follows: yellow color: score 0; marigold color: score 1; ochre color: score 2; bronze color: score 3.

PD-L1 expression in the peripheral tumor cells had a tendency to be higher than in the central tumor cells (Figure 3C, top left panel; *p* = 0.0625). We next stratified the data according to disease severity (tumor grade, primary tumor stage, and the presence of LN metastases) and revealed that disease severity did not correlate with PD-L1 expression in the central tumor cells (Figure 6A–C), nor did tumor grade significantly impact PD-L1 expression in the peripheral tumor cells (Figure 6D). However, the presence of LN metastases and tumor stage significantly correlated with the expression of PD-L1 in peripheral tumor cells (Figure 6E–F). These data showed not only that the severity of SGC correlated with PD-L1 expression in tumor cells but also that this correlated expression was clustered in the tumor periphery.

## 4. Discussion

This study showed that the PD-L1 and PD-1 molecular signatures are clustered predominantly in the periphery of SGC tumors and may reflect the disease severity.

Infiltration of tumors with TIICs has been shown to be a valuable marker of disease prognosis and sensitivity to immunotherapy [36,37,38]. In some tumors, high levels of TIICs are associated with a favorable prognosis [40], whereas in other tumors, they are associated with a poor prognosis [40,41]. Similar results have also been reported regarding their relationship to tumor resistance/sensitivity to immunotherapy [41]. In SGCs, the TIIC content largely differs among individual subtypes. However, it does not necessarily reflect the biologic behavior of the disease. For instance, adenoid cystic carcinoma and salivary duct carcinoma largely differ in the TIIC content [42], but they both belong to the most therapeutically challenging subtypes of SGC [43,44]. Therefore, the prognostic value of TIICs in SGCs needs to be strengthened by evaluating their molecular signatures. In this study, we showed that PD-L1^+^ and PD-1^+^ TIICs were differentially present in SGC tumors. However, the main difference was their distribution between the tumor center and periphery. Whereas PD-L1^+^ TIICs were evenly distributed between the tumor center and periphery, PD-1^+^ TIICs were largely located in the tumor periphery. Surprisingly, however, this accumulation did not reflect disease severity, indicating that peripheral PD-1^+^ TIICs alone are not good predictors of disease severity.

Studies have shown that PD-1 can also be expressed in tumor cells [39]. Our data confirmed the presence of PD-1^+^ tumor cells in several investigated tumors. Unexpectedly, our data also confirmed a connection between the presence of PD-1^+^ tumor cells in the tumor center and tumor grade. Very recent findings have indicated that the population of PD-1/PD-L1-expressing tumor cells is resistant to anti-PD-1/PD-L1 immunotherapy [39], and PD-1-expressing tumor cells block neutrophil cytotoxicity in cancer [45]. However, whether this mechanism of action of PD-1^+^ tumor cells is also at play in high-grade SGC tumors remains to be elucidated.

The expression of PD-L1 in tumor cells strongly indicates that tumor cells use the PD-L1/PD-1 signaling axis to suppress the immune system locally [46]. This local cell-mediated suppression may be at play in the tumor periphery to prevent tumor infiltration with tumor-targeting immune cells [35,36]. Our data indeed showed that large proportions of PD-L1-expressing tumor cells were clustered in the periphery of the investigated tumors. In addition, contrary to PD-1 expression in peripheral TIICs, where no correlations with disease severity were found, PD-L1 expression in peripheral tumor cells correlated with the presence of LN metastases and tumor stage. Surprisingly, the content of peripheral PD-L1^+^ tumor cells did not correlate with the content of peripheral PD-1^+^ TIICs (data not shown). Previous studies have shown that the exhaustion patterns in peritumoral immune cells are associated with good treatment response to checkpoint inhibitors [47,48]. Moreover, it has also been shown that peritumoral application of ICI is associated with improved survival in certain cancer types [49]. In accordance with these studies, our findings also suggest that PD-L1 expression in the peripheral TIICs of advanced SGCs could predict ICI treatment efficacy [50]. In addition, PD-L1 expression in the peripheral tumor cells of SGCs could represent a valuable tool for the prediction of disease prognosis.

The location of the immune and tumor cell signatures is an important factor related to biomarkers [36,37]. Attempts have been made to evaluate not only the intra-tumoral cell signatures but also the signatures that go beyond the tumors themselves [36,37,51,52]. However, there are also increasing attempts to evaluate the signatures within individual locations of the tumors themselves [53]. The results of our study revealed that the location of PD-1- and PD-L1-expressing cells plays an important role in shaping their predictive values and associations with the severity of SGC. The most striking finding in this study was the location-dependent values related to the expression of PD-L1 in peripheral tumor cells. Whereas PD-L1 expression in tumor cells located in the tumor center was not associated with the presence of LN metastases or tumor stage, PD-L1 expression in tumor cells located in the tumor periphery was. These findings showed that not only the para-tumoral compartments [52,54] but also the intra-tumoral compartments may provide additional information. Furthermore, this information can reveal new predictive values of biomarkers based on their tumor/immune signatures.

## 5. Conclusions

In this study, we, for the first time, showed the PD-L1 and PD-1 expression patterns in both the tumor cells and TIICs of SGC patients. A differential evaluation of the tumor center and tumor periphery across diverse histological subtypes of SGC revealed the role of peripheral TIICs and tumor cells in the understanding of the factors that dictate the disease severity. Moreover, the PD-1 expression in the peripheral TIICs of SGCs revealed a potential for the implementation of ICI immunotherapy in SCGs patients. The PD-L1 expression in the peripheral tumor cells of SGCs showed a significant association with disease severity. Our findings show that the periphery of SGC tumors represents a biomarker niche for evaluating SGC severity and signatures of tumor–immune system interplay.

## Figures and Tables

**Table 1:** Clinicopathological variables for the patient cohort. Shading stands for each group of variables and helps to easily locate the main variables.

	Patients (*n* = 62)	Patients (%)
Gender		
Female	36	58.06%
Male	26	42.94%
Histology		
Mucoepidermoid carcinoma	13	20.98%
Adenoid cystic carcinoma	11	17.74%
Acinic cell carcinoma	8	12.90%
Adenocarcinoma, not otherwise specified (NOS)	6	9.68%
Salivary duct carcinoma	6	9.68%
Undifferentiated carcinoma	4	6.45%
Carcinoma ex pleiomorphic adenoma	3	4.84%
Mammary analogue secretory carcinoma (MASC)	3	4.84%
Myoepithelial carcinoma	3	4.84%
Squamous cell carcinoma	2	3.23%
Adenosquamous carcinoma	1	1.61%
Carcinosarcoma	1	1.61%
Cribriform cystadenocarcinoma	1	1.61%
Grade		
Low (0)	22	35.48%
High (1)	40	64.52%
Primary tumor stage		
1	15	24.19%
2	9	14.55%
3	6	9.68%
4	31	50.00%
Not established	1	1.61%
Metastases		
Yes (+)	19	30.65%
No (–)	43	69.35%
Age		
≥45 years	13	20.97%
45–70 years	27	43.55%
≤70 years	22	35.48%

**Table 2:** Antibodies used for the immunohistochemical analysis.

Antibodies	Clone	Dilution	Source	Target Antigen Retrieval	Cells	Type of Positivity
PD-1	Rabbit monoclonal	1:200	Zytomed, Berlin, Germany	None	Tumor cells/Tumor-infiltrating immune cells	Plasmatic staining
PD-L1	Mouse monoclonal	1:50	Dako, Santa Clara, California	EnVision FLEX target retrieval solution, low pH	Tumor cells/Tumor-infiltrating immune cells	Membrane staining

## Supplementary Materials

The following are available online at https://www.mdpi.com/2227-9059/9/2/97/s1, Figure S1: Disease severity does not correlate with the expression of PD-L1 in TIIC.
