# Supplementary material for: The Periphery of Salivary Gland Carcinoma Tumors Reveals a PD-L1/PD-1 Biomarker Niche for the Evaluation of Disease Severity and Tumor—Immune System Interplay
Source: Biomedicines. 2021 Jan 20;9(2):97. doi: 10.3390/biomedicines9020097 (PMC7909271; doi:10.3390/biomedicines9020097)
Supplement: Supplementary file 1 [file biomedicines-09-00097-s001.zip › biomedicines-1031383-supplementary ZS Rev.docx]

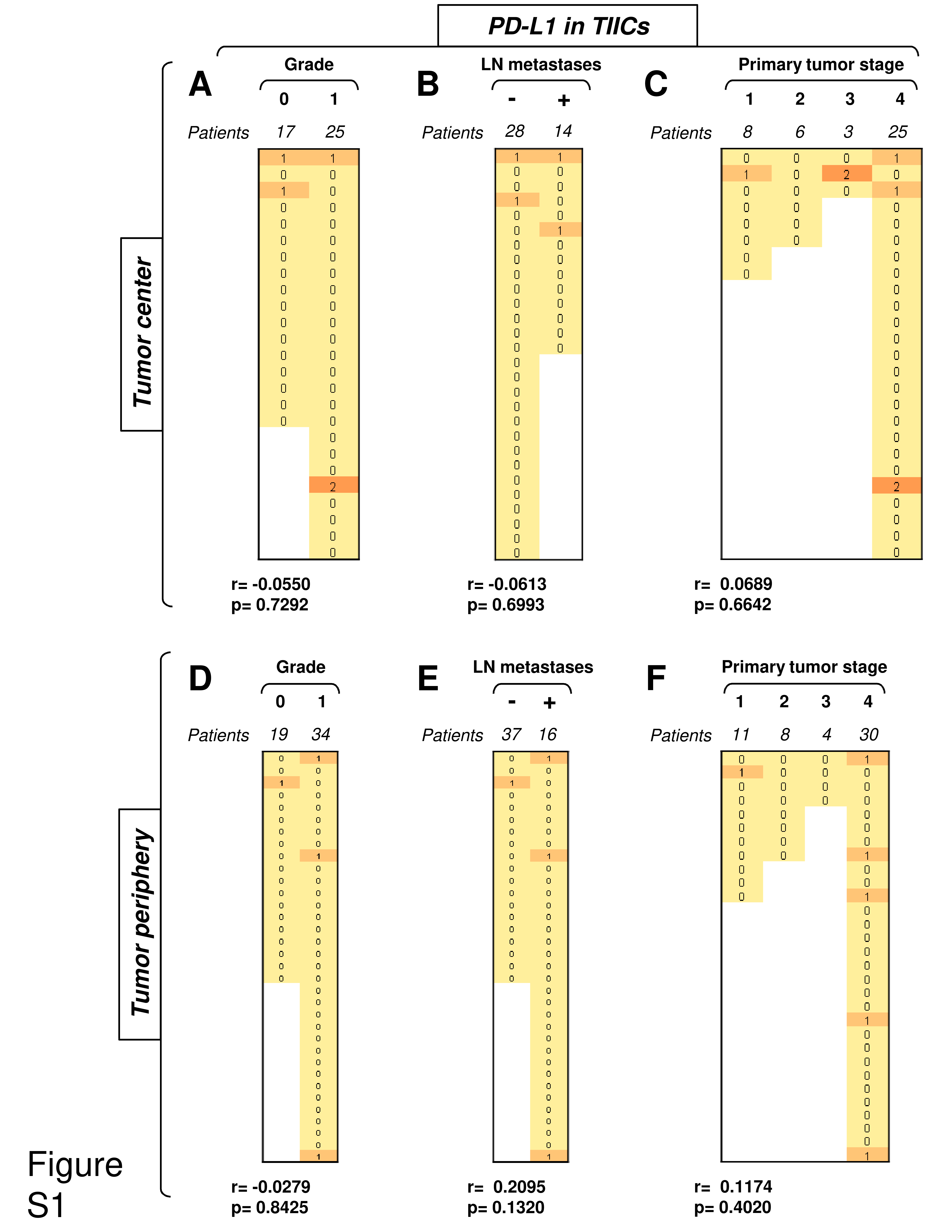


**Figure 1. Disease severity does not correlate with the expression of PD-L1 in TIICs.** (**A**) The cohort of 42 SGC patients was stratified into 2 groups according to tumor grade (grade 0 and grade 1), and the Spearman correlation with the expression of PD-L1 in TIICs in the tumor center was determined. (**B**) The patient cohort in **A** was stratified into 2 groups according to the presence (**+**) or absence (**-**) of LN metastases, and the Spearman correlation was determined as in **A**. (**C**) The patient cohort in **A** was stratified into 4 groups according to the primary tumor stage, and the Spearman correlation was determined as in **A**. (**D**–**F**) The cohort of 53 SGC patients was stratified into groups as in **A**–**C**, and the Spearman correlation with the expression of PD-L1 in TIICs in the tumor periphery was determined. In **A**–**F**, the correlations were evaluated by Spearman correlation tests (**A**–**C**: *n* = 42, **D**–**F**: *n* = 53). *P<0.05 was considered significant. In **A**–**F**, the expression analyses of PD-1 were performed according to the scoring system described in the Materials and methods section. The data are presented as a heat map with the scores.
