# Supplementary figures and images for: The Periphery of Salivary Gland Carcinoma Tumors Reveals a PD-L1/PD-1 Biomarker Niche for the Evaluation of Disease Severity and Tumor—Immune System Interplay
Source: Biomedicines. 2021 Jan 20;9(2):97. doi: 10.3390/biomedicines9020097 (PMC7909271; doi:10.3390/biomedicines9020097)

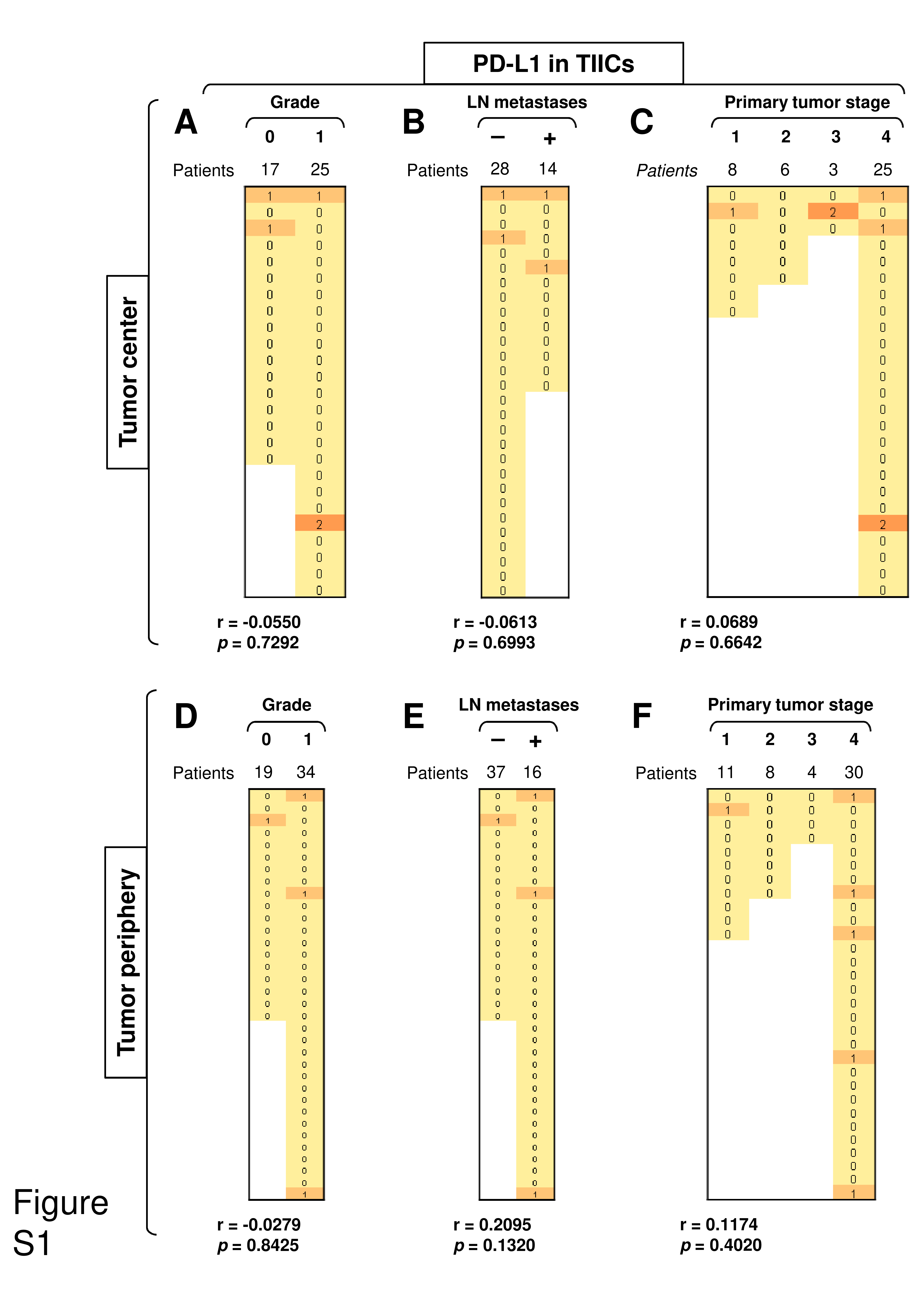

Supplement: Supplementary file 1 [file biomedicines-09-00097-s001.zip › proof-Supplementary Figure S1.tif]
